# Fake It ‘Till You Make It—The Pursuit of Suitable Membrane Mimetics for Membrane Protein Biophysics

**DOI:** 10.3390/ijms22010050

**Published:** 2020-12-23

**Authors:** Johannes Thoma, Björn M. Burmann

**Affiliations:** 1 Wallenberg Centre for Molecular and Translational Medicine, University of Gothenburg, 405 30 Göteborg, Sweden; johannes.thoma@gu.se; 2 Department of Chemistry and Molecular Biology, University of Gothenburg, 405 30 Göteborg, Sweden

**Keywords:** membrane protein, lipid bilayer, membrane mimetic

## Abstract

Membrane proteins evolved to reside in the hydrophobic lipid bilayers of cellular membranes. Therefore, membrane proteins bridge the different aqueous compartments separated by the membrane, and furthermore, dynamically interact with their surrounding lipid environment. The latter not only stabilizes membrane proteins, but directly impacts their folding, structure and function. In order to be characterized with biophysical and structural biological methods, membrane proteins are typically extracted and subsequently purified from their native lipid environment. This approach requires that lipid membranes are replaced by suitable surrogates, which ideally closely mimic the native bilayer, in order to maintain the membrane proteins structural and functional integrity. In this review, we survey the currently available membrane mimetic environments ranging from detergent micelles to bicelles, nanodiscs, lipidic-cubic phase (LCP), liposomes, and polymersomes. We discuss their respective advantages and disadvantages as well as their suitability for downstream biophysical and structural characterization. Finally, we take a look at ongoing methodological developments, which aim for direct in-situ characterization of membrane proteins within native membranes instead of relying on membrane mimetics.

## 1. Introduction

The overwhelming majority of scientific articles on membrane proteins introduces this class of proteins by mentioning their contribution of roughly 30% to organisms’ genomes, thus, highlighting their importance and resulting self-explanatory relevance. In the interest of avoiding a repetition of what has been written countless times before, let us instead spend a moment of consideration on the endangered species of the polar bear. The polar bear (*Ursus maritimus*) is perfectly adapted to life on the annual sea ice of the arctic circle, a very specific habitat, which it roams as wide-ranging predator hunting seals [1]. When polar bears are relocated from their natural environment to live in captivity in zoological gardens, they have a high tendency to develop abnormal repetitive behavior, such as stereotypical pacing and head nodding [2], and have severely increased infant mortality rates [3]. Similar alterations of behavior and infant mortality in captivity have been reported for several different species, whereupon the degree of these alterations is directly correlated to the extent of the environmental difference, but rarely as pronounced as in polar bears [3]. Much like this admittedly farfetched example, membrane proteins evolved to reside in the very specific amphipathic lipid bilayer environment of biological membranes and their removal from this environment often results in pronounced structural and functional ramifications [4,5,6]. It is, thus, one of the great challenges of membrane protein biophysics to characterize membrane proteins, while maintaining the specific nature of their lipid bilayer environment to be able study this class of proteins in a biologically meaningful context. The continuously progressing efforts to recreate this environment, in order to facilitate the biophysical characterization of membrane proteins are the subject of this review.

Lipid bilayers form the physical permeability barriers, which segregate cells and cellular compartments. Driven by the hydrophobic effect, amphiphilic lipid molecules self-assemble to form lamellar bilayers, with their hydrophobic moieties facing the core of the bilayer and their hydrophilic head groups facing the surrounding aqueous environment [7]. Cellular membranes are formed from a large variety of chemically very diverse lipids [8], ranging from hundreds of different lipid species in “simple” prokaryotic organisms like *Escherichia coli* to thousands in more complex eukaryotic organisms [9,10]. The diverse physiochemical properties of different cellular membranes are shaped by their lipid composition [9]. Biological membranes obtain their functionality only through the presence of specialized integral membrane proteins, which transmit molecules, energy and stimuli across these physical barriers. To fulfil these crucial functions membrane proteins depend on the properties of the surrounding membrane environment [11,12,13]. Factors, such as the lipid composition and bilayer asymmetry, membrane curvature, tension as well as the fluidity of the bilayer directly impact the structural and functional integrity of membrane proteins [13,14,15].

**Figure 1:**
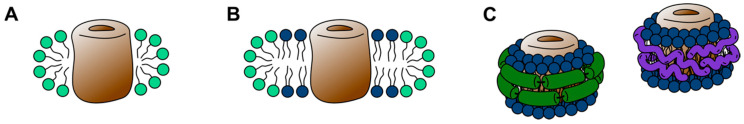
Solubilized states of membrane proteins. (**A**) Membrane protein in a detergent micelle (cyan). (**B**) Membrane protein in a disc-shaped bicelle containing lipids (blue). (**C**) Membrane proteins in nanodiscs stabilized by membrane scaffold protein (MSP, green) and amphipathic polymers (purple).

Unfortunately, in their native cellular form, membrane proteins immanently defy the requirements of biophysical experiments, which demand protein samples of high purity and high concentration, often in form of a solution. In addition to being insoluble, individual membrane protein species occur in rather low densities in cellular membranes. To bridge this gap and make membrane proteins experimentally accessible a multitude of membrane mimetics have been developed over the last decades. As implied by their name, membrane mimetics attempt to imitate the environment of lipid bilayers. In their most fundamental forms, as detergent micelles, this means simply to recreate the hydrophobic core environment of a lipid bilayer [16]. However, more complex forms such as bicelles and nanodiscs try to incorporate a certain number of lipid molecules (Figure 1). Moreover, purified membrane proteins can be reconstituted into bilayers of synthetic lipids or lipid extracts, attempting to closely resemble the original lipid bilayer a membrane protein was purified from.

Naturally, all these methods have their pros and cons and not all of them are compatible with different biophysical methods. For example, solution NMR (Nuclear magnetic resonance) spectroscopy, single-particle cryo-EM (electron microscopy), and X-ra/neutron solution scattering methods demand membrane proteins in a solubilized form, typically in the form of micelles, bicelles or nanodiscs. Solid-state-NMR, negative stain EM, and AFM (atomic force microscopy) are suitable for larger membrane assemblies such as proteoliposomes, whereas X-Ray crystallography requires samples in a three-dimensional (3D) crystalline form. The hydrophobic contacts required to maintain the structural integrity of membrane proteins within these crystals can either be satisfied by detergents or in case of lipidic cubic phases (LCP) by lipid molecules. In this review, we survey the currently available membrane mimetic systems, weigh their advantages, as well as their disadvantages, evaluate how they impact the structural and functional states of membrane proteins and assess their suitability for various biophysical methods.

## 2. Detergent Micelles

The archetype of membrane mimetics are detergents, which are routinely used to solubilize membrane proteins. Detergents are amphipathic molecules, which self-assemble to form micelles in aqueous solutions. Based on their molecular structure and properties, namely the charge of their hydrophilic head groups, detergent molecules can be subdivided into ionic, non-ionic, and zwitterionic detergents. Thereby, ionic detergents such as sodium dodecyl sulfate (SDS) are considered “harsh”, due to their (differently pronounced) denaturing effect on membrane proteins, ranging from minor structural alterations [17] to loss of function and complete unfolding [18]. Notably, some membrane proteins remain rather unaffected by ionic detergents. In particular, bacterial outer membrane proteins have shown to be very resistant to SDS denaturing, due to extensive hydrogen bonding networks stabilizing the transmembrane β-barrels of these proteins. In fact, the resulting altered migration behavior in SDS page is frequently used as an indicator of the folding state of outer membrane proteins [19,20].

For the solubilization of membrane proteins in a functional form more widely used are “mild” non-ionic detergents, such as Octyl-L-D-glucoside (OG) and Dodecyl-L-D-maltoside (DDM), which tend to retain the structural integrity of solubilized proteins and leave inter- and intra-molecular protein-protein interactions intact. The latter is particularly important for the solubilization of multimeric membrane protein complexes and especially DDM has proven quite useful for the purification of intact complexes [21,22,23,24]. The third group are zwitterionic detergents, such as Lauryldimethylamine-N-oxide (LDAO), the hydrophilic head groups of which have a positive, as well as a negative charge. While, the overall electrically neutral, zwitterionic detergents form an intermediate between ionic and non-ionic detergents, with a stronger solubilizing potential than non-ionic detergents and a less pronounced deactivating effect than ionic detergents [25].

**Figure 2:**
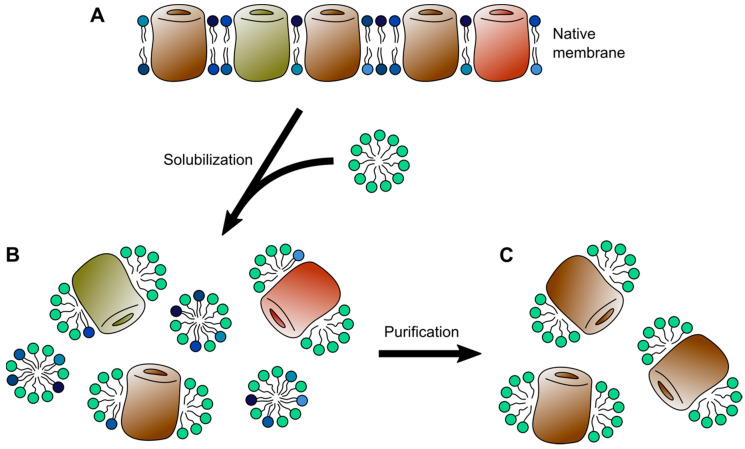
Solubilization and purification of membrane proteins. (**A**) Native membrane containing a membrane protein of interest over a background of other membrane proteins in a diverse lipid composition. (**B**) Mixed membrane protein-detergent and lipid-detergent micelles resulting from solubilization of the membrane through the addition of detergent. (**C**) Purified membrane protein in detergent micelles after removal of undesired lipid components and protein contaminations.

The solubilization of biological membranes usually occurs *via* two stages with increasing detergent concentration (Figure 2). Initially, at low detergent concentrations, the detergent molecules insert into the lipid bilayer, resulting in destabilization and fragmentation. At high concentration, typically exceeding the detergents critical micellar concentration (CMC), the lipid bilayer is dissolved, resulting in binary lipid-detergent or protein-detergent, as well as ternary lipid-protein-detergent mixed micelles [26,27]. Thereby different detergents vary in their capacity to solubilize different cellular membranes. For example detergents like Triton X-100 and Sarkosyl have been shown to selectively solubilize inner membranes of Gram-negative bacteria and mitochondria, while leaving their outer membranes largely unaffected [28,29]. Lipid microdomains (sometimes referred to as lipid rafts), which typically contain cholesterol and saturated sphingo- and glycerophospholipids in a liquid ordered phase, are resistant to mild detergents, such as Triton X-100, and thus, remain as detergent-resistant membranes after solubilization [30,31]. Likewise, the amount of endogenous lipid molecules bound to membrane proteins varies greatly depending on the detergent used to solubilize a membrane [32]. In this context, mass spectrometry and in particular tandem mass spectrometry (MS/MS) is a powerful method to characterize not only the binding of lipids to membrane proteins, but also their effects on membrane protein oligomerization [33,34]. The choice of detergent depends ultimately on the planned downstream biophysical characterization and especially detergents with a low CMC, despite effectively solubilizing most membranes, can be difficult to remove and are, thus, of limited suitability for methods requiring detergent removal [35].

The purification of a membrane protein from a cellular membrane typically begins with the isolation and solubilization of the membrane of interest [36]. Due to the differences in the way detergents interact with cellular membranes, their efficient solubilization involves screening for a suitable detergent [37]. Finding a detergent that also stably maintains a membrane protein and is suitable for downstream applications can require substantial screening work [38,39]. Once solubilized, the purification of membrane proteins follows similar principles as the routine purification of soluble proteins, relying on chromatographic methods including affinity, gel filtration, and ion exchange chromatography [36]. An alternative route to obtaining solubilized membrane proteins in a pure and folded state is refolding into detergent micelles. To this end, membrane proteins are transferred from a fully unfolded state in concentrated solutions of chaotropic salts into a detergent containing refolding buffer to adopt a folded state within the detergent micelles [40,41]. Interestingly, it was shown that in a similar way the cellular chaperone machinery can be exploited and refolding into micelles results in exactly the same folded protein state, regardless whether folding was initiated from a chaperone or chaotropic reagents [42]. Combined with recombinant protein expression in inclusion bodies, this method can expedite protein purification and can prove particularly useful when large amounts of a protein are required. However, despite routinely used, membrane proteins do not necessarily fold into a native structure and refolding can result in non-native multimers and structural intermediates [43,44].

Using NMR spectroscopy membrane proteins can be directly characterized in a detergent solubilized form, without the need for additional downstream modifications [45]. Particularly useful when analyzing membrane proteins with NMR spectroscopy is the use of deuterated detergents, which eliminate interfering proton signals originating from the detergent [46]. While, a handful of membrane protein structures have been determined using NMR spectroscopy [47,48,49,50], the real strength of the method lies in its ability to probe dynamic processes [51]. In the micellar state NMR spectroscopy determines the residue-specific dynamics, can probe interactions with ligands and detect conformational changes in solution [45,52]. Moreover, unlike other structural techniques, NMR spectroscopy can yield detailed information on highly dynamic and unstructured regions of membrane proteins, such as loops [53,54]. Therefore, NMR spectroscopy could capture the subtle differences imposed on the structural conformation of membrane proteins by different detergents [54]. In this context, it should be stressed that, despite often resulting in high-resolution structural information, detergent stabilized states of membrane proteins, especially in Dodecylphosphorylcholine (DPC), might often be non-functional states [55].

Actuated by the recent resolution revolution [56], single-particle cryo-EM emerged as another powerful technique to characterize membrane proteins in detergent micelles. Unlike NMR spectroscopy, which excels at characterizing small proteins, single-particle cryo-EM is best suited for large proteins and protein complexes [57]. Several membrane protein structures stabilized in detergent micelles could be solved by cryo-EM, including bacterial β-barrel assembly machinery (BAM) complex, mitochondrial TOM core complex, and the spinach cytochrome b_6_f complex [58,59,60]. However, despite efforts to streamline the preparation of membrane proteins for cryo-EM, the methodology is far from being routine work [61,62]. Detergent concentrations typically used in preparations of membrane proteins tend to interfere with the controlled formation of thin vitrified ice, often resulting in reduced image contrast [63]. Therefore, the preparation of membrane proteins for cryo-EM requires thorough removal of excess detergent [64]. Moreover, the surface to volume ratio of a sub-micrometer thin water film on an EM grid is much higher than in conventional liquid droplets and it is not yet fully clear how the consequentially altered fluid dynamics and the air-water interface impact solubilized macromolecules [61,65].

While, detergents arguably form a less than ideal environment for many membrane proteins their usage remains in most cases unavoidable. With very few exceptions, the solubilization of membrane proteins from the membranes of an expression host is commonly accomplished through the use of detergents [66,67]. The majority of membrane mimetics require the reconstitution of a membrane protein, which naturally requires the prior solubilization and purification of said membrane protein. Detergent micelles are therefore almost always the starting point for additional downstream applications involving more complex membrane mimetics.

## 3. 3D Crystals and Lipidic Cubic Phase

Despite a growing toolbox of alternative membrane mimetics, to date the majority of membrane protein structures have been solved by X-ray crystallography, utilizing 3D crystals of membrane proteins. The crystallization of detergent-stabilized solubilized membrane proteins follows similar methods and principles as the crystallization of soluble proteins based on vapor diffusion, microdialysis, and batch crystallization [68,69]. Crystals are, thereby, formed from a protein solution, supersaturated with precipitating agents such as salts or polyethylene glycol (PEG), which drive the aggregation of protein-detergent-complexes into ordered crystals [68]. Within these type II crystals the hydrophobic surfaces of membrane proteins remain satisfied through the co-crystallized detergent micelles, while the crystal lattice is preferentially formed by polar protein-protein interactions [70,71]. Successful crystallization often depends on the choice and nature of the detergent and high detergent concentrations or the use of detergents with a large micelle size can impair crystal formation [69].

Following the first high-resolution crystal structure of a membrane protein, the photosynthetic reaction center from *Rhodopseudomonas viridis* [72], a great variety of membrane protein structures could be solved using X-ray crystallography. These include groundbreaking structures such as bacterial potassium channel KcsA [73] and lactose permease LacY [74], which resulted in unprecedented insight into the molecular details of these proteins in particular and membrane proteins in general. Nevertheless, it should be noted that solving crystal structures of membrane proteins often only is possible due to substantial molecular engineering. This includes the introduction of mutations, which arrest proteins in a certain conformation [75]—an effect, which can also be achieved through the binding of ligands or antibody fragments [76,77]—deletions of parts of the proteins to eliminate unfavorable crystal contacts [78], or chimeric modifications of proteins [79]. In addition, ambiguities can inhere in crystal structures, imposed by the crystallization conditions, together with uncertainties in the identity and position of atoms and molecules within crystal structures [80]. Even similar sample preparations can lead to altered structures, as for example evidenced in the translocator protein (TSPO) that yielded two different α-helical bundles depending if the structure was determined in milder DM or DDM [81,82], compared to the harsher zwitterionic DPC [83] and it remains unclear whether the different structures represent simply alternative states of the protein. However, the structures, obtained in the milder detergents, are in better agreement with known functional mutations and the ligand binding site shows a larger degree of conservation [81].

A valuable alternative to detergent-micelle mediated 3D crystallization of membrane proteins is crystallization in the lipidic cubic phase (LCP) [84]. LCP takes advantage of the propensity of monounsaturated monoacylglycerols, such as monoolein, to form a bicontinuous cubic mesophase, a single lipid bilayer organized into a three-dimensional bilayer structure containing an aqueous channel system [85]. The cubic phase is formed spontaneously when the lipid is mixed with solubilized or dispersed protein solutions, while crystal formation is driven by the addition of a precipitant [86]. In addition to monoolein, LCP can contain various accessory lipids, which can either remain from co-purification with a membrane protein or can be specifically added during the crystallization process [87]. Through the stabilization in a lipid bilayer, LCP is thought to provide a more natural environment for membrane proteins and LCP has been successfully used, particularly with membrane proteins which have small polar surfaces, such as the seven-helix-bundles of rhodopsins [88,89,90] and G-protein-coupled receptors (GPCRs) [91,92].

One limitation of LCP crystallization is its propensity to result in microcrystals [85]. However, microcrystals grown in LCP are ideally suited to be studied using serial femtosecond crystallography (SFX) [93]. The latter utilizes ultrashort pulses generated by an X-ray free-electron laser (XFEL) [94] to sequentially collect data from a continuous stream of microcrystals [95]. By the short duration of highly intense X-ray pulses the crystals are vaporized before radiation damage can occur [94], thus facilitating the time-resolved characterization of dynamic structural transitions in crystallized proteins [96]. These studies have provided remarkable insight into the activation of different photoreceptors, by visualizing the so-called protein quake [97,98,99]. In addition to SFX, another method that has been show to provide high-resolution structural data of microcrystalline membrane proteins is so-called microcrystal electron diffraction (Micro ED) [100,101], which was recently applied to study the tetrameric sodium channel NaK in DDM based crystals [102] as well as the human adenosine A_2A_ receptor in LCP [103].

## 4. Bicelles and Nanodiscs

The first attempt to incorporate a substantial amount of lipids into solubilized membrane protein systems were bicelles. Bicelles (bilayered micelles; Figure 1) are formed by phospholipids, traditionally dimyristoyl-phosphatidylcholine (DMPC), in a planar discoidal bilayer assembly, which are surrounded by a scaffold of either a detergent, such as CHAPS, or short-chained lipids, such as dihexanoyl-phosphatidylcholine (DHPC) [104]. Thereby, bicelles can be designed to adopt a variety of shapes, ranging from small bilayer discs over wormlike structures to large perforated lamellar assemblies, dictated by the lipid-to-detergent, or longchain-to-shortchain lipid ratio, respectively [105,106]. In order to reconstitute membrane proteins into bicelles either a detergent-stabilized membrane protein is integrated into preformed bicelles or the bicelles can be formed through the addition of detergents to proteoliposomes that were assembled beforehand [106]. Through the incorporation of lipid molecules, bicelles have been shown to outperform micelles in their ability to maintain membrane proteins in a functional state [107,108]. However, it has also been shown that different lipid compositions of bicelles significantly influence membrane protein dynamics [109]. Moreover, molecular dynamics (MD) simulations indicate increased peptide solvation of transmembrane segments within small bicelles compared to larger bilayer systems [110].

While, originally developed as a membrane system for solid state NMR spectroscopy [111], bicelles found a broad audience in biophysics [106,112]. Due to the increased lipid content, bicelles are larger than most purely detergent-based micellar systems. Yet, their molecular tumbling permits detailed characterization of reconstituted membrane proteins by solution NMR spectroscopy [113,114]. By stabilizing the transmembrane region of the HIV-1 envelope spike (Env) in DMPC/DHPC bicelles the atomic resolution structure of the trimeric assembly could be determined [115,116,117], which remained elusive in the previous cryo-EM structure, possibly due to the detrimental influence of the DDM/sodium deoxycholate micelles [118]. Likewise, a direct comparison of the dimeric transmembrane domain of Glycophorin A in DPC micelles and DMPC/DHPC bicelles, respectively, showed reduced conformational fluctuation and enhanced stability of the transmembrane α-helixes in the bicellar lipid environment [119].

Bicelles have also been implemented as an alternative crystallization method trying to combine the incorporation of lipids, as used in LCP, with the facility of detergent based crystallization [120]. Bicelle crystallization exploits the temperature-dependent ability of lipid/amphiphile mixtures to exchange between different three-dimensional structural arrangements [121]. When this concept was initially introduced with studies of bacteriorhodopsin, the protein was found to be embedded in bicelles as a stable monomer instead of its usual trimeric assembly [120]. Bicelle-based 3D crystals have since these early studies successfully been used with multiple membrane proteins, including the human G-protein-coupled receptor (GPCR) β2 adrenergic receptor (β_2_AR) [122], and the eukaryotic mitochondrial voltage-dependent anion channel (VDAC) [123].

Evolving from bicelles, the recent years have seen great development in the field of nanodiscs. Collectively, the term nanodiscs refers to the lipid bilayer particles similar to discoidal bicelles, which are surrounded by a scaffolding molecule (Figure 1). Yet, the size-range in which scaffolded nanodiscs can be prepared is somewhat limited, compared to bicelles. Following the first account of a lipid nanodisc surrounded by membrane scaffold protein (MSP) [124], several other types of scaffolds have been described, including saposin proteins (salipro) [125], as well as copolymer-scaffolded nanodiscs utilizing styrene-maleic acid (SMA) and diisobutylene/maleic acid (DIBMA) [126,127]. The formation of nanodiscs follows similar principles as the formation of bicelles, starting from a mixture of detergent-solubilized lipid, detergent-solubilized protein, and MSP or saposin, respectively, and is driven by subsequent detergent removal [125,128,129].

Unlike protein-based nanodiscs, which require the reconstitution of membrane proteins from a detergent-solubilized micellar state, co-polymers have shown certain detergent-like properties. When mixed with membrane preparations, these polymers can extract “native nanodiscs” containing membrane proteins together with a fraction of the lipid molecules surrounding the proteins [130,131]. While, nanodiscs certainly have the ability to preserve the local lipid composition around a membrane protein, they cannot maintain membrane asymmetry [132]. The latter is due to the dynamic nature of the different nanodisc systems. On the one hand equilibration between both leaflets can occur when lipid molecules flip around the edges of the nanodisc and on the other through diffusional as well as collisional transfer. Polymer-scaffolded nanodiscs have been shown to readily exchange proteins, lipids, and polymer components at much higher rates compared to MSP-scaffolded nanodiscs or unilamellar lipid vesicles [133,134]. In contrast, MSP-scaffolded nanodiscs have been found to exhibit internal lipid dynamics, which are comparable to lipids in liposomes [135].

Nanodiscs have been widely used with a wide range of biophysical methods, including NMR spectroscopy [136,137,138], electron microscopy [139,140] and atomic force microscopy [141]. Therefore, the use of nanodiscs has proven successful even with complex systems. For example, individually MSP-nanodisc-stabilized inner and outer membrane components of bacterial tripartite efflux pumps MexAB–OprM and AcrAB–TolC could be recombined to form stable intact complexes bearing two separate nanodiscs [142]. The open pore state of bacterial Tc toxin complex TcdA1, which could not be sufficiently stabilized in Tween-20 detergent micelles or liposomes [143,144], could be resolved in high detail embedded in nanodiscs [145], whereas the corresponding crystal structure could only reveal a closed pre-pore state [143]. Likewise, when characterized in nanodiscs, aforementioned HIV envelope protein Env showed substantial differences in its arrangement and orientation to the membrane surface, compared to micellar and bicellar environments [146].

Direct comparisons of membrane proteins in micellar, bicellar and nanodisc systems revealed distinct differences between these membrane mimetics. For example, the bacterial outer membrane protein OmpX has been shown to form a stable β-barrel in all three environments, however, the dynamics and molecular motion differed substantially between the mimetics [147,148]. Similarly, the comparison of outer membrane protein BamA in LDAO micelles, DMPC/DHPC bicelles, and MSP-bounded DMPC nanodiscs suggested altered dynamics between the three environments [149]. Likewise, the α-helical tetrameric potassium channel KcsA has been shown to have reduced stability in nanodiscs, indicating altered dynamic properties, compared to DDM micelles [150]. In contrast, the CC-chemokine receptor 5 (CCR5), a GPCR which is inherently instable in detergents, such as DDM, could be stabilized in nanodiscs for prolonged NMR studies [151]. The human anion channel VDAC showed nearly identical folds in micelles, bicelles, and nanodiscs, however, unlike the first two, nanodiscs incorporated not only monomeric VDAC, but multimeric states, similar to the ones observed in native membranes [152].

## 5. Liposomes (and Polymersomes)

**Figure 3:**
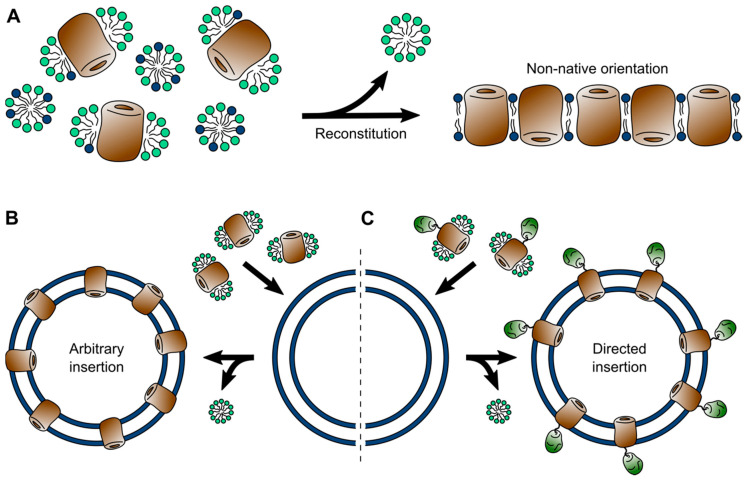
Reconstitution of membrane proteins into liposomes. (**A**) Bottom-up assembly from a ternary mixture (left), driven by detergent removal, typically results in an alternating up-down orientation of membrane proteins (right). (**B**) Undirected insertion of membrane proteins into preformed liposomes. (**C**) Directed insertion of membrane proteins into preformed liposomes with soluble domains facing outwards.

Native lipid bilayers contain a diverse blend of membrane proteins, interweaved with additional components, such as lipoproteins and carbohydrates in the form of glycoproteins and glycolipids [7]. In most native membranes any membrane protein of interest is vastly outnumbered by these additional components, making it inaccessible to biophysical investigations. Few exceptions, such as the purple membranes from Halobacteria [153,154] or disc membranes from vertebral rod cells [155], in fact allow proteins to be studied in native membrane isolates, which are covered in the next section. For the vast majority of membrane proteins, the alternative lies in the bottom-up reconstruction of an artificial lipid membrane. To this end, a membrane protein of interest is solubilized and isolated from the membrane of a suitable expression system using detergents and, naturally, the same principles and limitations of detergent micelles apply as covered above [156,157]. Following purification, in a subsequent step, membrane proteins are reconstituted into an artificial lipid bilayer. Reconstitution is generally achieved *via* one of two alternative routes (Figure 3). In one method the self-assembly of a lipid bilayer is driven by detergent removal from a ternary mixture of micelles containing the protein and separately detergent-solubilized lipids [158]. Alternatively, the solubilized membrane protein can be inserted into preformed liposomes [159]. In both cases assembly and membrane insertion are driven by the removal of the detergent and can be achieved by several means, all aiming at reducing the detergent concentration (far) below the CMC. Thereby, the method of detergent removal can have a strong influence on the resulting proteoliposomes. Dialysis can result in homogeneous vesicles, but is time-consuming and limited to detergents with high CMC. More rapid methods, such as size exclusion chromatography, rapid dilution, and the use hydrophobic adsorbents often result in inhomogeneous protein distribution and in-complete detergent removal [35].

Self-assembled lipid bilayers allow better control over the lipid-to-protein ratio and thus can result in a very dense protein packing within the bilayer, culminating in a 2D crystalline assembly [160,161]. However, due to the rotational freedom during assembly, membranes formed this way typically contain membrane proteins in a non-native up-down configuration, with either half of the proteins inserted in the bilayer in opposite directions (Figure 3). While random orientation can occur as well during insertion into preformed liposomes, asymmetry between the two solvent accessible poles of a membrane protein, for example in membrane proteins containing a soluble domain, can bias insertion with the soluble domain facing outwards (Figure 3) [159,162,163]. In fact, by fusing a soluble domain to one side of a membrane protein, this phenomenon can be utilized to drive unidirectional insertion of the protein into liposomes [164].

Naturally, a reconstituted artificial lipid bilayer cannot fully reassemble the complexity of a native membrane and features like membrane asymmetry or local variations in the lipid composition, sometimes referred to as lipid rafts, are impossible to mimic. Nevertheless, since artificial bilayers allow precise control over the lipid composition they are ideal proxies for detailed studies on how different lipid composition of a membrane affect membrane proteins. The utilization of reconstituted lipid bilayers for example allowed detailed characterization of lipid-induced topological switches in proteins of the major facilitator family [165,166], which could be confirmed in vivo [167]. Similarly, liposomes of varying lipid composition are a valuable tool to understanding membrane protein folding at a molecular level. Therefore, not only the folding process from chemically denatured states is of interest [168,169], but much focus has been laid on chaperone-dependent protein folding [170,171].

Until challenged by the advent of single particle cryo-EM, two-dimensional (2D) crystalline membrane protein assemblies were the system of choice for structure determination by EM [172,173]. Unlike X-Ray crystallography, which requires 3D crystals that are sometimes difficult to obtain from membrane proteins, electron crystallography is ideally suited for the 2D crystals of membrane proteins. Electron crystallography revealed the first high resolution structures of Bacteriorhodopsin [174], Aquaporin [175], and light-harvesting complex [176]. A rather recent development is the use of protein-containing liposomes in cryo-electron tomography combined with subtomogram averaging for the high-resolution reconstruction of membrane proteins [177,178,179]. Moreover, liposomes have been increasingly used to investigate membrane proteins using solid-state NMR spectroscopy. Recent advances in fast and ultra-fast (>100 kHZ) magic angle spinning (MAS) solid-state NMR spectroscopy based on ^1^H-detection resulted in enhanced sensitivity and resolution comparable to solution NMR, required to facilitate high resolution NMR studies of membrane proteins [180,181]. Solid-state NMR spectroscopy has, for example, been used to determine structural the details and conformational rearrangements of α-helical transmembrane proteins such as KcsA reconstituted in lipid bilayers [182,183], as well as bacterial β-barrel outer membrane proteins such as OmpA and OmpG [184,185]. Importantly, all of these structures revealed nuanced differences, such as structural alterations and altered dynamics compared to previously reported structures.

Another powerful tool for characterizing the membrane proteins reconstituted in lipid bilayers is atomic force microscopy (AFM), due to its ability to be operated in liquid environments at physiological temperatures [186,187]. Adsorbed to atomically flat surfaces, such as muscovite mica or highly oriented pyrolytic graphite (HOPG), AFM allows imaging of the membrane topographies of solid-supported planar lipid bilayers at molecular resolution [188,189,190]. AFM could reveal ligand-induced conformational changes of membrane proteins [191], details on the electrostatics of their accessible surfaces [192,193] and their dynamic behavior in lipid bilayers [194,195]. Moreover, AFM allows the direct physical manipulation of individual membrane proteins, facilitating detailed studies of their behavior under force [196,197,198], their folding behavior [199,200,201], as well as their interaction with tethered ligands [202].

In addition to being used for the structural characterization of membrane proteins, lipid bilayers allow the functional examination of membrane proteins, in particular molecular transport phenomena, which cannot be probed in solubilized states. Therefore, electrophysiological measurements, either based on fused liposomes or utilizing black lipid membranes, allow detailed characterization of diffusion through transmembrane pores as well as probing their electrochemical properties [203,204,205,206]. Similarly, molecular transport is routinely studied in intact liposomes based on osmotic swelling. The latter is particularly powerful when combined with fluorogenic reactions conducted inside the liposomes lumen in stopped-flow experiments [207,208].

The ability of artificial lipid bilayers to maintain membrane proteins in a functional state is showcased by their use as a bottom-up platform for the assembly of nanocells in synthetic biology, whereby membrane proteins are embedded in liposomes to create functional systems with potential applications as nano-sized reaction compartments, drug delivery vehicles and novel therapeutics [209,210,211]. In this context, a biomimetic alternative to lipid bilayers are membranes formed from amphiphilic block copolymers [212,213,214]. While the physical properties of these are very different from lipids, their bilayer forming abilities are driven by the same principles, and polymersomes can effectively maintain membrane proteins in a functional form [215,216]. In fact, in mixed polymer-lipid bilayers under certain conditions membrane proteins have been shown to preferentially reside within the polymer rather than the lipid fraction, depending on their relative fluidity [217,218].

## 6. Native Membranes

Whereas so far no mimetic entirely met the physiological requirements of membrane proteins, native membranes, in the majority of cases, cannot meet the experimental requirements of the modern biophysicist. Native membranes are notoriously difficult to handle. Typically, they contain a particular protein of interest only in small quantities over a much larger background of other membrane proteins. Nevertheless, native membrane preparations from *E. coli*, also known as Kabackosomes, were used to characterize molecular transport through membrane proteins long before the first membrane protein structures were uncovered [219,220]. Moreover, some specialized cellular membranes natively contain high densities of distinct membrane proteins, which are sufficiently pure to permit biophysical studies directly in the unaltered native membranes. The best example are the purple membranes from archaea such as *Halobacterium salinarum* and *Halobacterium halobium*, which naturally contain bacteriorhodopsin in a 2D crystalline form [154]. In fact, the first structural models of bacteriorhodopsin were determined using native purple membranes isolated from *H. halobium* [221]. While later preparations employing increased 2D crystals, obtained through detergent treatment of purple membranes, resulted in models with increased resolution [174,222], direct comparison of detergent treated crystals to native membranes showed that lipid boundaries separating individual trimers within the native membranes were removed [223]. A similar approach, based on partial de-lipidation was used to observe crystalline assemblies of porins in situ, in bacterial outer membrane sacculi [224].

While these early electron crystallographic studies required 2D crystalline assemblies of membrane proteins, advancements in cryo-electron tomography nowadays allow direct in situ analysis of membrane proteins in non-crystalline native membranes [225,226]. Although, data on integral membrane proteins so far remains sparse, the method has already proven its potential. Aided by membrane targeting nanoparticles, active preprotein-carrying TOM–TIM23 supercomplexes could be localized and subsequently visualized in situ in yeast mitochondrial membranes [227]. Intriguing examples showcasing the potential of cryo-electron tomography are the in situ structures of envelope spanning bacterial secretion systems, such as the structure of an intact primordial type III secretion system, which could be determined in *Chlamydia trachomatis* elementary bodies [228]. Other examples are the in situ structures of AcrAB–TolC efflux pump in intact *E. coli* cells [229], the type IV secretion system in intact *Legionella pneumophila* cells [230], as well as the membrane complex of a type VI secretion system in *E. coli* [231]. A very promising approach to study membrane proteins in situ is the enrichment of secreted extracellular vesicles with specific proteins, which could be exploited to study integral type I membrane proteins from *C. elegans* as well as Herpes simplex virus [232].

In addition to electron microscopy, solid-state NMR spectroscopy has been successfully employed to study membrane proteins embedded in native membranes. In this context, native *Escherichia coli* inner membranes were used as a proxy to characterize Anabaena sensory rhodopsin (ASR), which was found to form hexagonal packing in DMPC/DMPA liposomes but a square lattice assembly in the *E. coli* membrane [233]. Furthermore, solid-state NMR spectroscopy was used to characterize bacterial BAM complexes in native outer membranes, suggesting increased structural disorder in the native environment [234]. Recently, a combination of cryo-electron tomography and of ^1^H-detected solid-state NMR spectroscopy was employed to reveal different conformations of the bacterial membrane protein YidC in native membranes [235].

One exceptional method to study native membranes is AFM, which does not depend on the preparation of highly homogeneous samples [236,237]. The ability of AFM to imaging native membranes at molecular resolution was initially demonstrated with native purple membranes. Following initial topographies, which allowed discrimination of individual proteins [238], AFM was optimized to reveal the submolecular details of single bacteriorhodopsin molecules in great detail [239]. Using AFM, two-dimensional arrays of aquaporin 0 (AQP0) could be observed in native lens core membranes, surrounded by densely packed gap junction channels, and AQP0 array formation could be followed using time-lapse AFM [240]. Similarly, AFM could reveal native supramolecular assembly of VDAC in dense and sparse domains in native yeast mitochondrial outer membranes [241] as well as the closely packed, paracrystalline dimeric arrangement of rhodopsin arrays in native mouse disc membranes [242]. More recently, AFM was used to distinguish small and large protruding proteins in dimeric photosystem II oxygen-evolving complexes within native spinach grana membranes [243]. In addition to high-resolution imaging, AFM-based single-molecule force spectroscopy (SMFS) was used to probe the force response of individual membrane proteins in native membranes [196]. In this context, protein-enriched outer membrane vesicles from *Escherichia coli* were recently employed to reveal subtle differences in the dynamics of outer membrane proteins between native membranes and reconstituted lipid bilayers [244,245].

## 7. Conclusions and Future Perspectives

The last several decades have seen an enormous increase in efforts to optimize membrane mimetics to facilitate the structural and functional characterization of membrane proteins using biophysical methods leading to a constantly growing toolbox of diverse options (Table 1). Multiple membrane proteins could be characterized in several different mimetics, allowing the direct comparison and revealing the influence of the different mimetics on the structure and dynamics of these membrane proteins. Whereas for some membrane proteins these different tools are all in good agreement, for others vast differences have been observed, imposed by the respective mimetics used. For some membrane proteins, only a small set of mimetics could stabilize their folded state sufficiently to permit biophysical characterization, whereas other mimetics let to destabilization, impairing detailed characterization. Only in very few cases could membrane proteins be characterized in their native bilayer environment, some of which only revealed subtle, possibly negligible differences to mimetic systems, whereas others were substantially impacted by the mimetic environment.

Unlike the case of the polar bear, which can in fact be observed in its natural environment, as well as in captivity, thus, allowing a direct comparison between the two environments, for the majority of membrane proteins observations embedded in the native lipid environment are until now inexistent. Despite decades of studying membrane proteins with exceptional effort and the development of a plethora of groundbreaking sophisticated methods, we have only caught the first glimpses providing snapshots of their structural details within artificial environments. For many membrane proteins, these studies yielded priceless insight into their molecular architecture, which are supported by a multitude of functional investigations in vitro, as well as in vivo, yet, their behavior under native cellular conditions remains elusive. Clearly, to close this gap much more research on membrane proteins embedded in their native environment is necessary in the future. The recent trend towards in situ membrane protein biophysics will certainly help to illuminate this blind spot and provide in-depth insight into the details underlying membrane protein function.

## Figures and Tables

**Table 1:** Pros and cons of different membrane systems.

System	Pro	Contra	Suitable methods
Detergent micelles	Universally used; starting point for downstream applications	Can have denaturing effects; may disrupt complexes; de-lipidation of membrane proteins	Single-particle Cryo-EM; solution NMR; X-ray / neutron solution scattering; MS/MS
3D crystals	Most prevalent system for structure determination; can include lipids (LCP)	Non-native crystal contacts; often requires protein engineering; proteins are “locked” in one state; crystallization artifacts	X-ray crystallography; Micro-ED
Bicelles	Lipid system; can be used for 3D crystallization; variety of shapes and sizes	Limited lipid diversity; altered lipid dynamics	Solution NMR; solid-state NMR; (X-ray crystallography)
Nanodiscs	Lipid system; broad range of lipid compositions; possible to extract native lipid composition	Limited size range; altered lipid dynamics; membrane asymmetry is lost	Single particle Cryo-EM; solution NMR; Cryo-electron tomography; AFM
Liposomes	Lipid system; Broad range of lipid compositions; high protein density possible; facilitate transmembrane transport studies	Often non-native protein orientation; not possible to create asymmetric bilayers	Electron crystallography; Cryo-ET; solid-state NMR; AFM; electrophysiology; fluorometry
Native membranes	Native environment	Often difficult to handle; low content of protein of interest over “contaminants”	Cryo-ET; solid-state NMR; AFM

## Abbreviations

AFM	Atomic force microscopy
AQP0	Aquaporin 0
ASR	Anabaena sensory rhodopsin
β_2_AR	β2 adrenergic receptor
BAM	β-barrel assembly machinery
CCR5	CC-chemokine receptor 5
CMC	Critical micellar concentration
DM	Decyl-L-D-maltoside
DHPC	Dihexanoyl-phosphatidylcholine
DIBMA	Diisobutylene/maleic acid
DMPC	Dimyristoyl-phosphatidylcholine
DDM	Dodecyl-L-D-maltoside
DPC	Dodecylphosphorylcholine
EM	Electron microscopy
ET	Electron tomography
GPCRs	G-protein-coupled receptors
HOPG	Highly oriented pyrolytic graphite
LDAO	Lauryldimethylamine-N-oxide
LCP	Lipidic cubic phases
MAS	Magic angle spinning
MS	Mass spectrometry
MSP	Membrane scaffold protein
MicroED	Microcrystal electron diffraction
MD	Molecular dynamics
NMR	Nuclear magnetic resonance
OG	Octyl-L-D-glucoside
PEG	Polyethylene glycol
SDS	Sodium dodecyl sulfate
SFX	Serial femtosecond crystallography
SMA	Styrene-maleic acid
SMFS	Single-molecule force spectroscopy
Salipro	Saposin lipo-protein
XFEL	X-ray free-electron laser
VDAC	Voltage-dependent anion channel
